# Circulatory cytokeratin 17, marginal zone B1 protein and leucine-rich α2-glycoprotein-1 as biomarkers for disease severity and fibrosis in systemic sclerosis patients

**DOI:** 10.11613/BM.2022.030707

**Published:** 2022-10-01

**Authors:** Paul Bălănescu, Eugenia Bălănescu, Cristian Băicuș, Anca Bălănescu

**Affiliations:** 1 Internal Medicine Department, University of Medicine and Pharmacy Carol Davila, Bucharest, Romania; 2 Clinical Immunology Laboratory CDPC, Colentina Clinical Hospital, Bucharest, Romania; 3 Clinical Research Unit RECIF (Reseau d’Epidemiologie Clinique International Francophone), Bucharest, Romania; 4 Pediatrics Department, University of Medicine and Pharmacy Carol Davila, Bucharest, Romania

**Keywords:** systemic sclerosis, marginal zone B1 protein, leucine-rich α2-glycoprotein-1, cytokeratin 17, fibrosis

## Abstract

**Introduction:**

Systemic sclerosis (Ssc) is a multiorgan debilitating autoimmune disease that associates the triad: vascular involvement, tissue fibrosis and profound immune response alterations. Numerous previous studies focused on identification of candidate proteomic Ssc biomarkers using mass-spectrometry techniques and a large number of candidate Ssc biomarkers emerged. These biomarkers must firstly be confirmed in independent patient groups. The aim of the present study was to investigate the association of cytokeratin 17 (CK17), marginal zone B1 protein (MZB1) and leucine-rich α2-glycoprotein-1 (LRG1) with clinical and biological Ssc characteristics.

**Material and methods:**

Serum CK17, MZB1 and LRG1 were assessed in samples of the available Ssc biobank comprising of samples from 53 Ssc patients and 26 matched age and gender controls.

**Results:**

Circulatory CK17, LRG1 and MZB1 concentrations were increased in Ssc patients. Cytokeratin 17 is independently associated with Ssc disease activity. Patients with pulmonary fibrosis expressed higher LRG1 and MZB1 concentrations. Serum MZB1 concentrations were also associated with extensive skin fibrosis.

**Conclusions:**

Serum CK17, MZB1 and LRG1 were confirmed biomarkers for Ssc. LRG1 seems a good biomarker for pulmonary fibrosis, while MZB1 is a good biomarker for extensive skin fibrosis. CK17 proved to be independently associated with Ssc disease severity, higher CK17 values being protective for a more active disease.

## Introduction

Systemic sclerosis (Ssc) is a multiorgan debilitating autoimmune disease that associates the triad including vascular involvement, tissue fibrosis and profound immune response alterations (*1*). In order to improve treatment and monitoring approaches of these patients, clinicians need additional prognostic tools (*2*). Biomarkers that are easy to quantify in the clinical laboratory setting and are available from biological samples obtained from minimally invasive procedures could improve Ssc management. Proteomic biomarkers available in blood are easy to be identified in any clinical immunology laboratory using relatively cheap enzyme-linked immunosorbent assays (ELISA) without extensive training of the staff. Numerous proteomic biomarkers were evaluated for Ssc patients but the clinical utility of most of these biomarkers is still limited (*3*). In addition, there are numerous previous studies that focused on identification of candidate proteomic Ssc biomarkers using mass-spectrometry techniques from a wide array of biological samples (*4*, *5*). From all these studies a large number of candidates Ssc biomarkers emerged. However, in order to be included in current clinical practice, these biomarkers must firstly be confirmed in independent patient groups and validated for current clinical practice in clinical trials with superior study design. Among these candidate biomarkers, cytokeratin 17 (CK17), marginal zone B1 protein (MZB1) and leucine-rich α2-glycoprotein-1 (LRG1) seem promising due to their physiopathological role in fibrosis and inflammation.

Cytokeratin 17 is an alarmin and its main characteristic is that its expression in pluristratified epithelium is faint in the absence of an injury, but it rapidly increases in inflammatory cutaneous lesions (either infectious or autoimmune, *e.g.* psoriasis) (*6*). Serum CK17 concentrations also proved to be a prognostic biomarker in cervical cancer, higher CK17 being associated with a poorer prognosis (*7*). Cytokeratin 17 plays a pivotal role in wound healing *via* keratinocyte proliferation (*8*). Also, CK17 is involved in the immunomodulatory shift between Th1/Th17 and Th2 responses, as it has been showed *in vivo* that absence of CK17 is associated with Th2 immune response (*9*).

Marginal zone B1 protein is a co-chaperone localized in the endoplasmic reticulum (ER) and expressed solely by marginal zone B lymphocytes in the final process of differentiation into plasma cells. In addition, MZB1 mediates lymphocyte adhesion and migration *via* vascular cell adhesion protein 1. Alongside with other ER chaperones like heat shock protein 90 (Hsp90), MZB1 is important for correct maturation of IgM molecules (*10*).

Leucine-rich α2-glycoprotein-1 is a pro angiogenetic protein that also exerts anti-fibrotic properties, as high LRG1 serum concentrations were also identified in patients with end stage kidney disease due to renal fibrosis (*11*).

The aim of the present study was to investigate the association of CK17, MZB1 and LRG1 with clinical and biological Ssc characteristics. To our knowledge, this is the first study that evaluated circulatory CK17, MZB1 and LRG1 with clinical characteristics of Ssc patients.

## Material and methods

### Subjects

In this study, serum samples from the already available Ssc biobank of the Internal Medicine Clinic of Colentina Clinical Hospital were used. Data about the inclusion of samples in the biobank, evaluation of the included patients and descriptive data of patients are detailed elsewhere (*12*). Briefly, the Ssc biobank includes samples of 53 Ssc patients (4 males/49 females, median age 56 (25-81) years) and 26 healthy age and gender matched controls (2 males/24 females, median age 52 (26-63) years). The healthy control group consisted of patients that referred to Colentina Clinical Hospital but did not have any inflammation nor record of previous Raynaud’s phenomenon. All patients had extensive clinical, biological and imagistic evaluation in order to determine the severity of the disease. This study was performed on the same available Ssc biobank as our previous study and are part of the same research grant. All participants in the study signed an informed consent. The study was approved by the local ethics committee (No. 10/2020) and it was conducted according to the Declaration of Helsinki.

### Methods

Briefly, patients had their pulmonary function evaluated by chest X-ray, high resolution computed tomography (hr-CT), flow spirometry, diffusing capacity for carbon monoxide (DLCO) and transthoracic Doppler echocardiography (for determination of pulmonary hypertension, PHT). Frequency of Raynaud’s phenomenon, presence of calcinosis, digital ulcers, telangiectasias, sclerodactyly and calculation of total modified Rodnan skin score was recorded. Severity of disease was evaluated using European Scleroderma Trials and Research group (EUSTAR) criteria (*13*).

Serum CK17 concentrations were evaluated using a commercial ELISA kit (Human CK-17/KRT17, Elabscience Biotechnology, Houston, USA) according to manufacturer instructions. The intra-assay coefficient of variation stated by the manufacturer was 10%. The inter-assay coefficient of variation stated by the manufacturer was 10%. Limit of detection for CK17 was 0.16 ng/mL.

Serum MZB1 concentrations were evaluated using commercial ELISA kit (Human MZB1, RayBiotech, Peachtree Corners, USA) according to manufacturer instructions. The intra-assay coefficient of variation stated by the manufacturer was 10%. The inter-assay coefficient of variation stated by the manufacturer was 12%. Limit of detection for MZB1 was 3.65 pg/mL.

Serum LRG1 concentrations were evaluated using commercial ELISA kit (Human LRG1, RayBiotech, Peachtree Corners, USA) according to manufacturer instructions. The intra-assay coefficient of variation stated by the manufacturer was 10%. The inter-assay coefficient of variation stated by the manufacturer was 12%. Limit of detection for LRG1 was 0.3 ng/mL. All ELISA kits were “Research use only” and there was no internal control available.

All samples were analysed in duplicate and mean optical density was used for concentration determination according to standard curve. Optical densities were evaluated using DynaRead 5206 plate reader (Dynex, Buštěhrad, Czech Republic) and all calculations were performed in AlaDyn software (Prague, Czech Republic).

### Statistical analysis

Normal distribution was evaluated using the Shapiro-Wilks test. Continuous variables were summarized as median and interquartile range (IQR). Nominal variables were summarized as ratio. In order to evaluate differences between continuous variables non-parametric tests were used (Mann Whitney U test). Correlation between continuous variables were evaluated using Kendall-tau coefficient. For covariate adjustment, logistic regression was used when dependent variable was binary. Statistical analysis was performed using IBM SPSS Statistics 27 software for Mac OS (IBM Corp., Armonk, USA). Statistical significance was set at P < 0.05.

## Results

Table 1 summarizes the main characteristics of Ssc patients and demographic data of controls.

**Table 1:** Characteristics of systemic sclerosis patients and healthy controls

**Patient characteristics**	**Systemic sclerosis patients (N = 53)**	**Healthy controls (N = 26)**	**P**
Age, years	56 (25-81)	52 (26-63)	0.060
Female gender	49/53	24/26	1.000
Disease duration (months)	84 (95)	-	N/A
lSSc	20/53	-	N/A
dcSSc	33/53	-	N/A
Modified Rodnan score	15 (17)	-	N/A
ILD presence	28/53	-	N/A
%DLCO	78% (22)	-	N/A
PHT presence	7/53	-	N/A
PAPs (mm Hg)	25 (4)	-	N/A
% predicted FVC	97% (20)	-	N/A
Raynaud’s phenomenon	53/53	-	N/A
Daily Raynaud’s phenomenon	37/53	-	N/A
Digital ulcer presence	12/53	-	N/A
Digital ulcer number	0 (2)	-	N/A
Sclerodactyly presence	38/53	-	N/A
Telangiectasia presence	27/53	-	N/A
Calcinosis presence	10/53	-	N/A
Oesophageal hypomotility	31/53	-	N/A
CRP (mg/L)	3.5 (7.3)	-	N/A
ESR (mm 1 hour)	16 (17)	-	N/A
Low complement level	6/53	-	N/A
EUSTAR score severity index	3 (3)	-	N/A
Steroid therapy	30/53	-	N/A
Calcium channel blocking agents	16/53	-	N/A
ACE inhibitors	11/53	-	N/A
Continuous variables are presented as median and interquartile range while categorical variables are presented as ratio. lSSc - localized systemic sclerosis. dcSSc - diffuse systemic sclerosis. ILD - interstitial lung disease. %DLCO – percent diffusing capacity for carbon monoxide. PHT - pulmonary hypertension. PAPs – pulmonary arterial pressure. FVC - forced vital capacity. CRP – C-reactive protein. ESR - erythrocyte sedimentation rate. ACE - angiotensin convertase enzyme. N/A- not applicable.			

### Serum CK17 in Ssc patients

Serum cytokeratin 17 concentrations were significantly higher in Ssc patients compared to healthy controls (1.38 ng/mL (0.83) *vs* 0.64 ng/mL (0.70), P < 0.001).

Patients with frequent (daily) Raynaud’s episodes had higher circulatory CK17 compared to patients without daily Raynaud’s episodes. Patients with sclerodactyly, telangiectasia, PHT and active Ssc disease had lower circulatory CK17 concentrations compared to Ssc patients without the clinical characteristic (Table 2). In fact, serum CK17 independently associated with active Ssc disease in a logistic regression model that included ESR, DLCO, modified Rodnan skin score and low complement concentrations (either low C3 or low C4), (R^2^ = 0.71, Table 3).

**Table 2:** Comparison of circulatory CK17 in systemic sclerosis patients with and without specific clinical characteristic

**Clinical characteristic**	**CK17 in Ssc patients with specific clinical characteristic (ng/mL)**	**CK17 in Ssc patients without specific clinical characteristic (ng/mL)**	**P**
Daily Raynaud’s episodes	1.57 (0.96)	0.85 (0.43)	0.017
Sclerodactyly	1.20 (0.81)	1.89 (1.14)	0.050
Telangiectasia	1.10 (0.81)	1.67 (1.05)	0.015
PHT	0.76 (0.22)	1.57 (0.82)	0.015
Active Ssc disease (DAS ≥ 3, EUSTAR)	1.16 (0.76)	1.63 (1.37)	0.032
Data are presented as median and interquartile range. SSc - systemic sclerosis. PHT - pulmonary hypertension. DAS – disease activity score. EUSTAR - European Scleroderma Trials and Research group. CK17 - cytokeratin 17.			

**Table 3:** Logistic regression model for active systemic sclerosis as dependent variable

**Variable**	**OR (95% confidence interval)**	**P**
CK17 concentration	0.04 (0 to 0.56)	0.017
ESR	1.17 (1.02 to 1.34)	0.022
DLCO	1.05 (0.97 to 1.15)	0.200
Low complement concentration	0.042 (0 to 1.67)	0.090
Modified Rodnan score	1.30 (1.08 to 1.56)	0.004
(R^2^=0.71) OR - odds ratio. ESR - erythrocyte sedimentation rate. DLCO - diffusing lung capacity for carbon monoxide. A low complement concentration was defined as having either low C3 or low C4 fractions. CK17 - cytokeratin 17.		

Interestingly, serum CK17 concentrations were not different between patients with extensive *vs* localized cutaneous fibrosis (P = 0.700). Also, no differences between limited Ssc (lSSc) *vs* diffuse Ssc (dcSSc) patients were noted (P = 0.740).

Serum CK17 concentrations showed week but statistically significant correlation with pulmonary arterial pressure (PAPs) (r = - 0.31, P = 0.004) and with DLCO (r = 0.26, P = 0.015).

### Serum MZB 1 in Ssc patients

Serum MZB1 concentrations were higher in Ssc patients compared to healthy controls (236.43 ng/mL (142.62) *vs* 130 ng/mL (73.12), P < 0.001). Patients with Ssc with extensive skin fibrosis, presence of daily Raynaud’s phenomenon, dcSSc and pulmonary fibrosis had higher MZB1 concentrations compared to Ssc patients without the clinical characteristic (Table 4). A weak positive correlation was noted between MZB1 and modified Rodnan skin score (r = 0.26, P = 0.01).

**Table 4:** Comparison of circulatory MZB1 concentrations in systemic sclerosis patients with and without specific clinical characteristic

**Clinical characteristic**	**MZB1 in Ssc patients with specific clinical characteristic (ng/mL)**	**MZB1 in Ssc patients without specific clinical characteristic (ng/mL)**	**P**
High modified Rodnan skin score (> 14 points)	257.51 (148.82)	182.31 (125.97)	0.016
Ssc disease subtype	lSSc 172.05 (127.93)	dcSSc 250.10 (143.51)	0.035
Daily Raynaud’s episodes	239.10(162.60)	149.04 (94.56)	0.007
Pulmonary fibrosis	268.62 (144.64)	173.19 (95.78)	0.002
Active Ssc disease (DAS ≥ 3, EUSTAR)	244.60 (141.11)	184.54 (152.53)	0.056
Long disease duration (over 3 years)	222.82 (144.65)	223.11 (179.24)	0.054
Data are presented as median and interquartile range. lSSc - limited Ssc. dcSSC - diffuse Ssc. DAS – disease activity score. MZB1 - marginal zone B1 protein. EUSTAR - European Scleroderma Trials and Research group.			

### Serum LRG1 in Ssc patients

Serum LRG1 concentrations were statistically significant higher in Ssc patients compared to controls (40.88 ng/mL (20.72) *vs* 34.24 ng/mL (18.06), P = 0.012). Patients with pulmonary fibrosis had higher LRG1 concentrations compared to patients without pulmonary fibrosis (46.37 ng/mL (24.84) *vs* 30.27 ng/mL (15.79), P = 0.002). Serum LRG1 was weakly correlated with higher ESR (r = 0.45, P < 0.001), and higher PAPs (r = 0.25, P = 0.023) and negatively correlated with higher DLCO (r = - 0.25, P = 0.014). However, after adjustment for gender and Ssc subtype, LRG1 but not MZB1 were independently associated with pulmonary fibrosis in Ssc patients (Table 5, R^2^=0.54)

**Table 5:** Logistic regression model for pulmonary fibrosis in systemic sclerosis patients as dependent variable

**Variable**	**OR (95% Cl)**	**P**
Gender	0.27 (0.017 to 4.44)	0.370
Ssc subtype	0.12 (0.02 to 0.81)	0.029
LRG1	1.09 (1.01 to 1.15)	0.025
MZB1	1.00 (0.99 to 1.02)	0.079
(R^2^=0.54) OR-odds ratio. SSc – systemic sclerosis. MZB1 - marginal zone B1 protein. LGR1 - leucine-rich α2-glycoprotein-1.		

## Discussion

This study confirmed circulatory CK17, LRG1 and MZB1 as biomarkers for Ssc, as their serum concentrations were significantly higher compared to controls. In addition, association of these biomarkers with various clinical Ssc characteristics were described.

Lower CK17 was associated with active Ssc disease and with various clinical characteristics that are associated with more severe disease (sclerodactyly, telangiectasia and PHT). Previous fundamental proteomic studies indicated CK17 as candidate biomarker in Ssc patients, but confirmation studies were needed in order to assess its diagnosis and prognosis capacity (*5*, *14*). Although higher CK17 concentrations were noted in Ssc patients compared to healthy controls, lower CK17 concentrations associated poorer prognosis within Ssc group. This intriguing aspect could be explained by the fact that CK17 had a weak but negative correlation with disease duration and thus patients with recent disease have higher CK17 concentrations. Patients that already have established fibrosis after longer disease course would express lower CK17 concentrations. Circulating CK17 concentrations seem protective for more active disease phenotype (OR = 0.04, 95% CI 0.03 to 0.56, P = 0.017), probably *via* immune modulation. Cytokeratin 17 plays an important role in preventing Th1/Th17 switch to Th2 phenotype and it has been very recently proved that a prominent Th2 response is associated with active Ssc disease (*15*). Additional validation prospective studies that also include patients with very early Ssc are needed in order to evaluate circulatory CK17 as prognosis Ssc biomarker.

This study confirmed circulatory MZB1 as biomarker in Ssc patients. It also associated with extensive skin fibrosis, dcSSc, presence of daily Raynaud’s phenomenon and pulmonary fibrosis. To the best of our knowledge this was the first study to confirm MZB1 as biomarker for Ssc patients. Marginal zone B1 protein was also described as biomarker for other autoimmune diseases like systemic lupus erythematosus (SLE) or rheumatoid arthritis (RA). Higher MZB1 expression was associated with more active SLE disease or with a more severe rheumatoid syndrome in RA patients (*16*). As an interesting aspect, expression of MZB1 was also found on active autoimmune sites of inflammation other than lymph nodes like iris tissue or aqueous humour (*17*). Unfortunately, in our study no skin or pulmonary biopsies were taken from patients and as such one could not evaluate its presence in these sites. However, although MZB1 is an intracellular stress chaperone, ER chaperones were also found to be expressed extracellularly and in circulation due to necrosis or apoptosis and this is a possible explanation for its circulatory origin (*18*). As a stress chaperone, MZB1 was found to be associated with fibrosis in skin and lungs due to persistent inflammation that is mainly mediated by autoantibodies. Additionally, MZB1 proved to be a good biomarker for pulmonary fibrosis diagnosis in Ssc patients. These findings confirm previous observations and suggest a role of MZB1 in fibrosis development in Ssc patients (*14*). Likewise, MZB1 seems to be an attractive therapeutic target that would have some obvious advantages. Firstly, as MZB1 is only expressed by marginal zone B lymphocytes, its blocking with monoclonal antibodies would be associated with fewer adverse reactions. Future studies should consider evaluation of MZB1 expression in Ssc lung and skin as well in order to evaluate MZB1 as potential Ssc therapeutic target.

Leucine-rich α2-glycoprotein-1 concentrations were higher in Ssc patients compared to healthy controls. It seems that locally, LRG1 inhibits secretion of TGF-beta and proinflammatory cytokines (*11*). A similar pattern would also be observed in Ssc lungs but evaluation of LRG1 expression from pulmonary specimens is needed to confirm this hypothesis. Nevertheless, *in vivo* studies on murine induced Ssc models indicated that LRG1 is involved in both pulmonary and skin fibrosis development (*19*). Also, higher circulatory concentrations of LRG1 were previously noted in small group of Ssc patients but due to small sample size associations with clinical Ssc patterns were not possible. Data from our study suggest that LRG1 may be a biomarker for pulmonary but not for cutaneous fibrosis (as no associations were found with modified Rodnan scores or with presence of excessive cutaneous fibrosis). On the other hand, MZB1 was associated with both pulmonary and cutaneous fibrosis in univariate analysis but after adjustment for gender, disease subtype and LRG1, this association with pulmonary fibrosis presence was not significant, suggesting that LRG1 is a better biomarker for pulmonary fibrosis. This finding is also supported by the significant albeit weak correlations found between serum LRG1 and pulmonary function tests, as MZB1 did not show such correlation.

While also a weak positive correlation between LRG1 and ESR was found, LRG1 was not associated with Ssc disease severity. The correlation with ESR is not surprising, as LRG1 is induced in inflammation (*20*). Although LRG1 was found to be associated with decreased pulmonary function and active inflammation, it did not prove to be a good biomarker for Ssc disease activity.

The present study comprises a series of limitations. Firstly, the sample size is rather small, due to the fact that Ssc is a rare disease and also to the fact that the study was monocentric. Secondly, there are no follow-up data for the patients and therefore the prognostic capacity of the biomarkers could not have been appropriately evaluated. Nevertheless, to our knowledge this is the first study in the literature that confirmed these biomarkers in Ssc patients and that evaluated the associations with clinical Ssc features, as this is a first and necessary step in order to determine relevant biomarkers that had been identified in mass spectrometry studies.

## Conclusions

Serum CK17, MZB1 and LRG1 were confirmed biomarkers for Ssc. As for the associations with Ssc clinical features, LRG1 proved to be a good biomarker for pulmonary fibrosis, while MZB1 proved to be a good biomarker for extensive skin fibrosis. Cytokeratin 17 was independently associated with Ssc disease severity, higher CK17 values being protective for a more active disease. Future studies are needed to validate these biomarkers in current clinical practice.

## Data Availability

The data generated and analysed in the presented study are available from the corresponding author on request.
